# Optic Nerve Infiltration in Systemic Lymphoma in an HIV Patient

**DOI:** 10.7759/cureus.18041

**Published:** 2021-09-17

**Authors:** Pierre Rodriguez, Binav Baral, Kriti Ahuja, Muhammad Tariq, Maryam Zia

**Affiliations:** 1 Internal Medicine, John H. Stroger, Jr. Hospital of Cook County, Chicago, USA; 2 Hematology and Medical Oncology, John H. Stroger, Jr. Hospital of Cook County, Chicago, USA

**Keywords:** diffuse large b cell lymphoma, human immunodeficiency virus, lymphoma, optic nerve, optic neuritis, high dose methotrexate

## Abstract

Different mechanisms have been proposed in lymphomatous involvement of the optic nerve. They include isolated optic nerve lymphoma, optic nerve lymphoma associated with primary central nervous system (CNS) lymphoma, or with systemic lymphoma. We present one case of non-Hodgkin lymphoma of the optic nerve in a Human Immunodeficiency Virus (HIV) patient and discuss the mechanism of metastasis, classification of optic nerve involvement with clinical and radiologic features as well as treatment options. Despite the uncommon nature of optic nerve infiltration by lymphoma, prompt evaluation should be considered in patients with a history of lymphoma and visual symptoms as delays in treatment can result in permanent vision loss. The recommended initial workup includes neuroimaging and cerebrospinal fluid evaluation. Treatment options are not standardized but include intravenous and intrathecal chemotherapy, corticosteroids, and radiation.

## Introduction

Non-Hodgkin lymphoma (NHL) is the seventh most common cancer in both men and women. It is more common in the presence of advanced HIV infection and is almost always of B-cell origin. AIDS-related lymphomas include Burkitt lymphoma, diffuse large B-cell lymphoma (DLBCL), AIDS-related primary central nervous system lymphoma (PCNSL), primary effusion lymphoma, plasmablastic lymphoma, Kaposi's sarcoma-associated herpesvirus (KSHV)-associated multicentric Castleman’s disease, and classic Hodgkin’s lymphoma. In the combination antiretroviral therapy (ART) era, the incidence of AIDS-related lymphoma has decreased; however, the incidence is still higher compared to patients without HIV/AIDS. HIV-associated DLBCL and PCNSL tend to present with severe immunosuppression and are often linked to Epstein-Barr virus (EBV) infection [1]. DLBCL is the most common AIDS-related lymphoma and commonly presents with advanced disease and extranodal compromise.

## Case presentation

A 51-year-old Hispanic male presented with left facial pain and edema and was ultimately diagnosed with Hodgkin lymphoma, nodular sclerosis type, by anterior and posterior left buccal mucosa biopsy. PET scan showed extensive fluorodeoxyglucose (FDG)-avid lymphadenopathy within the neck, chest, abdomen, and pelvis consistent with the known diagnosis. He completed one cycle of ABVD in December 2019. One month later, he presented with rapid enlargement of the left testicle. Due to the high standardized uptake values (SUV) of the solid hypoechoic left testicular mass, left-sided orchiectomy was performed with pathology revealing high grade diffuse large B-cell lymphoma germinal center type, c-Myc positive with positive fluorescence in situ hybridization (FISH) result for Myc rearrangement; suggestive of 14q or IGH rearrangement, negative for BCL6 rearrangement and IGH/BCL2 fusion, t(14;18), for which he completed six cycles of DA-EPOCH-R with intrathecal methotrexate for central nervous system (CNS) prophylaxis. At that time, a bone marrow biopsy showed a normocellular marrow with no abnormal lymphoid aggregates or granulomas. Immunostains for CD3, CD20, and CD138 as well as kappa and lambda in situ hybridization (ISH) and Epstein-Barr encoding region (EBER) ISH were performed and interpreted as negative for involvement by a large B-cell lymphoma or Hodgkin lymphoma. Verbal and written consent were obtained from the patient to discuss this case report.

**Figure 1:**
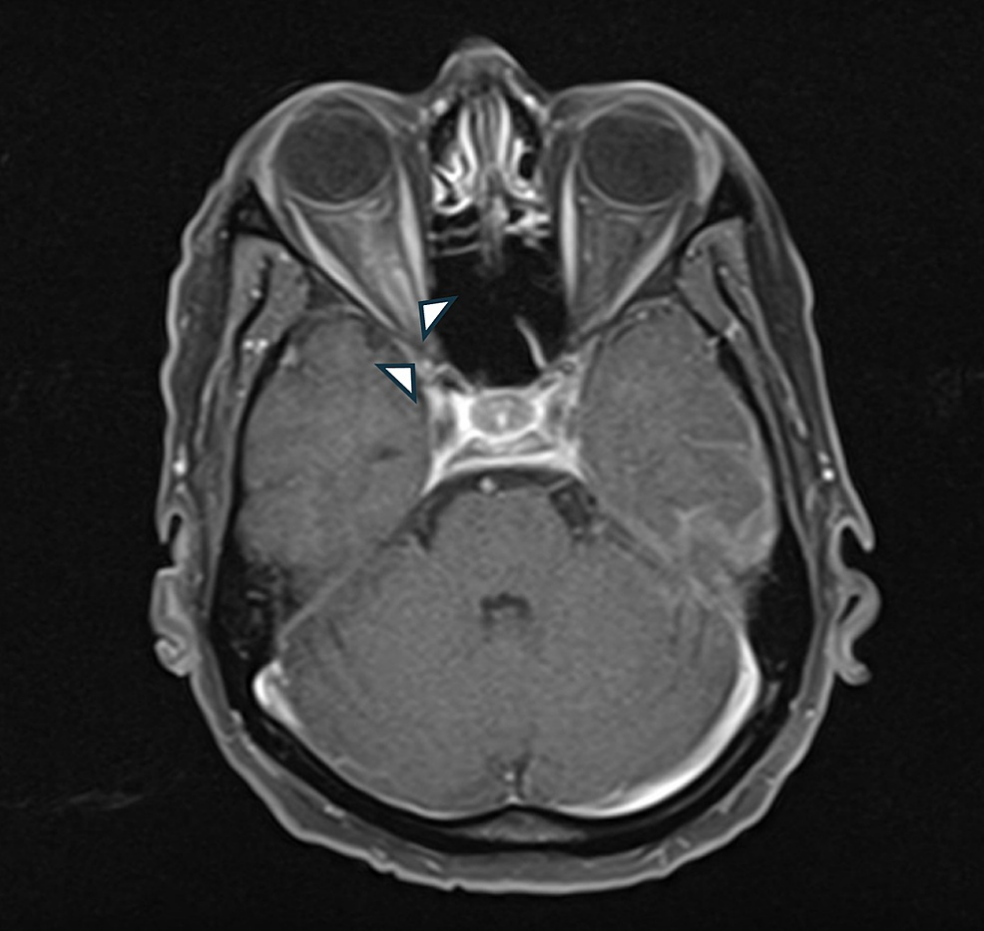
Arrowheads: axial T1-weighted gadolinium-enhanced MRI demonstrates contrast enhancement of the pre-chiasmatic and canalicular and orbital parts of the right optic nerve

**Figure 2:**
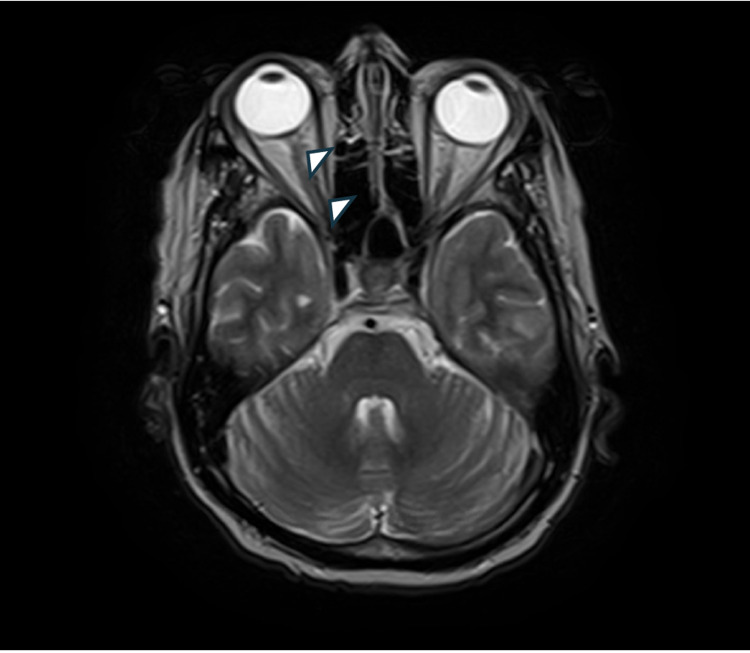
Arrowhead: axial T2-weighted gadolinium-enhanced MRI demonstrates hyperintense signal with heterogeneous enhancement along the right optic nerve sheath

One month after completing six cycles of DA-EPOCH-R with intrathecal methotrexate for CNS prophylaxis, he presented to the emergency department with worsening painless right blurry vision which progressed to complete vision loss associated with periorbital headache. He had no previous ophthalmologic history. On initial examination, he had visual acuity to hand motion in the right eye and a relative afferent pupillary defect (RAPD). Dilated fundoscopic examination of the right eye showed optic disk edema and retinal pigment epithelium atrophy without hemorrhage, microaneurysms, and anterior vitreous detachment (AVD) in the right eye. Magnetic resonance imaging (MRI) of the orbits was suspicious for lymphomatous involvement of the right optic nerve including slight asymmetric thickening of the canalicular and orbital segments of the right optic nerve as well as asymmetric hyperintense short tau inversion recovery (STIR) signal and hyperenhancement involving the pre-chiasmatic and canalicular segments of the right optic nerve (Figures 1, 2).

MRI of the brain showed abnormal enhancement of the left temporal leptomeninges, pituitary infundibulum, and the dura along the left tentorium suggestive of meningeal carcinomatosis as well as abnormal enhancements in the ependyma of the lateral and third ventricles. Lumbar puncture was performed and yielded cerebrospinal fluid (CSF) with an abnormal population of B-cells with CD19, CD20, uniform CD10 expression with monoclonal kappa surface light chain immunoglobulin expression, consistent with CSF involvement by the patient’s known diffuse large B-cell lymphoma. CSF was negative for bacteria and fungi. A systemic infectious disease evaluation was negative.

After MRI and CSF evaluation, the patient was started on corticosteroids and high-dose IV methotrexate with improvement in his right eye symptoms.

## Discussion

Optic nerve involvement can be further classified into primary intraocular lymphoma, PCNSL, and optic nerve involvement with metastatic CNS disease.

Primary intraocular lymphoma implies malignant lymphoid cells invading the retina, vitreous or optic nerve head with or without CNS involvement [2]. It commonly presents in older patients as chronic uveitis which is often unresponsive to corticosteroids treatment. The most common finding on ocular examination is vitreitis. CT of the head frequently shows isodense or hyperdense lesions. MRI shows lesions that are hypodense on T1-weighted and hypodense on T2-weighted images. Diagnostic vitrectomy and CSF are often necessary as imaging, although they play an important role in diagnosis, cannot always confirm the diagnosis [3].

Primary CNS lymphoma (PCNSL) is an uncommon entity accounting for roughly <0.7% of all malignant lymphomas and <1% of intracranial tumors [4], however, it can present more frequently in immunocompromised hosts including those living with advanced HIV and AIDS and organ transplant recipients on chronic immunosuppressive agents. In immunocompetent individuals, this entity is rare and if found, patients are frequently older than 60 years. The vast majority of PCNSL are B-cell in origin (DLBCL). It usually involves the brain parenchyma near the ventricles [5].

Patients with PCNSL develop neurologic signs over weeks and are not limited to focal neurological deficits, mental status changes, and behavioral changes but also intracranial hypertension syndrome with vomiting and papilledema on ophthalmoscopy [6]. On imaging, PCNSL is identified as a CT hyperdense enhancing supratentorial mass, with MRI T1 hypointense, T2 iso to hypointense, vivid homogeneous enhancement and restricted diffusion, little vasogenic edema, and no central necrosis.

Optic nerve involvement with CNS disease is a rare and aggressive disease. It presents with secondary CNS involvement in patients with systemic lymphoma. Malignant lymphoma cells may reach the CNS by hematogenous spread, direct invasion from contiguous structures, or extension along nerves. MRI with gadolinium contrast shows leptomeningeal, dural, subependymal, and cranial nerve involvement with thickening and T2 hyperintense enhancing portions which can be smooth, irregular, or nodular.

Role of neuroimaging

MRI is the preferred modality as it best characterizes the meningeal, cerebrospinal fluid, and axonal portions of the optic nerve. Typical findings include enlargement of the optic nerve, enhancement of the nerve sheath (MRI with gadolinium contrast), or tram-track sign (parallel thickening and enhancement around the optic nerve) [7]. These findings must be correlated with the history of the patient and clinical suspicion, as they can be present in other inflammatory or infectious processes involving the optic nerve including multiple sclerosis, primary optic nerve tumors such as meningioma, and optic glioma, sarcoidosis, syphilis, or tuberculosis. In patients who are unable to undergo MRI, CT imaging can be considered.

Lumbar puncture, CSF analysis, and optic nerve biopsy

CSF analysis is pivotal in the diagnosis of lymphomatous involvement of the optic nerve. It is unclear how many lumbar punctures are necessary in patients who are suspected to have developed CNS lymphoma; however, it is often enough for diagnosis and precludes the need for an optic nerve biopsy. Routine CSF studies include cell counts, protein, glucose, and lactate dehydrogenase (LDH). Glucose is often very low in patients with CNS lymphoma. Cytology, when combined with flow cytometry, is the gold standard for diagnosis. Flow cytometry is also important as it assists with the prognosis and identification of patients who might develop drug resistance [3]. Flow cytometry is commonly positive for CD19, CD20, CD22, CD79a, Ki-67, and light chain Ig (K or I). Although less common, flow cytometry can also be positive for CD23, CD25, CD53, and CD103. EBV has been associated with CNS lymphoma and therefore, polymerase chain reaction (PCR) has been used as a diagnostic tool to identify such cases in HIV or immunosuppressed patients, however, the positive predictive value is lower when differentiating lymphoma from CNS toxoplasmosis [8].

Optic nerve biopsy is reserved for patients who have progressive and significant vision loss despite appropriate empiric treatment or when lumbar puncture and CSF studies are indeterminate for diagnosis of CNS lymphoma and no etiology is identified for the optic neuropathy. Routine optic nerve biopsy is not necessary in most cases and it should be done by a specialist as it carries significant risk to patients who maintain visual acuity regardless of lymphomatous involvement of the optic nerve [3]. Methods used to get optic nerve tissue include image-guided fine-needle aspiration biopsy (FNAB), transconjunctival biopsy, and neurosurgical approach (open craniotomy).

Treatment

Although there is no consensus in treatment, most patients are treated with high-dose IV methotrexate, intrathecal methotrexate, intravenous corticosteroids, and radiation therapy. In patients with AIDS-related lymphoma, combination ART is warranted as higher CD4 and lower international prognosis index (IPI) have been independently associated with improved overall survival. Rituximab use is controversial in HIV-DLBCL patients. Even though rituximab has not been associated with a high toxic-death rate, it has also not been associated with improved overall survival [9] [10], except in adults with Burkitt lymphoma where dose-adjusted EPOCH-R showed effectiveness regardless of age and HIV status [11]. Our patient responded well after a high dose of IV methotrexate and corticosteroids.

## Conclusions

Patients with history of NHL presenting with optic neuritis should have evaluation for lymphomatous infiltration of the optic nerve. When signs and symptoms are concerning for optic nerve lymphoma, CSF evaluation with cytology and flow cytometry and neuroimaging including MRI with contrast are warranted. HIV is associated with increased risk of developing DLBCL. Our case supports hematogenous dissemination in patients with systemic NHL. Optic nerve biopsy is not routinely indicated except in patients who have failed empiric therapy or those with high index of suspicion for optic nerve lymphoma in the presence of negative diagnostic studies.
